# Effect of orbital manifestations among type 2 diabetes mellitus patients

**DOI:** 10.6026/973206300210529

**Published:** 2025-03-31

**Authors:** Gargi Mohan Achwal, Karambelkar V.H, Joshi B.S

**Affiliations:** 1 Department of Ophthalmology, Krishna Institute of Medical Sciences, Krishna Vishwa Vidyapeeth (Deemed To Be University), Karad, Maharashtra, India

**Keywords:** Diabetes mellitus, lifestyle modifications, orbital manifestation, patient counseling, cataract

## Abstract

Diabetes mellitus affects around 240 million people globally and it is projected to rise about 370 million by 2030. Therefore, it is of interest to evaluate the orbital and anterior segment orbital manifestation among 91 type 2 diabetes mellitus patients for ocular and general examination. The distribution of symptoms was cataract (71%), blepharitis (24%), dry eye (24%), mucormycosis (12%), cranial nerve palsies at 11%, recurrent changes in refraction (10%), primary open-angle glaucoma (5%), recurrent stye (4%), corneal ulcer (3%), iridocyclitis (3%), orbital cellulitis (2%), rubeosis iridis (1%), and neovascular glaucoma (1%) respectively. Thus, effective diabetes management should encompass patient counseling, nutritional guidance, lifestyle modifications, stringent blood glucose control and evaluation of treatment effectiveness and adherence.

## Background:

Complications related to diabetes mellitus that impact the anterior segment of the eye, such as the cornea, conjunctiva, and lacrimal glands, are often overlooked, even though they represent a major contributor to blindness in developed nations [1]. A lot of attention is paid to diabetic retinopathy, but it's important to remember that conditions affecting the anterior segment, like damage to corneal nerves and epithelial cells, are key in many other conditions [2]. Some of these are dry eye disease, corneal erosion, persistent epithelial defects, cataracts, uveitis, neovascular glaucomas, refractive changes, orbital cellulitis, muscle palsies and corneal ulcers that could be dangerous to your sight [3]. The implications of these complications highlight the extensive influence of diabetes on eye health, extending beyond just the posterior segment. Several of these conditions may encompass diabetes mellitus as a significant risk factor, contributing to the complications associated with them [4]. Early diagnosis of these conditions and their prompt management, coupled with intensive diabetes mellitus control under the guidance of a diabetologist, can effectively prevent numerous vision-threatening complications [5]. Diabetes mellitus and otic relation can lead to morbidity and mortality [6]. In another study, diabetes mellitus pandemic had also shown its impact on orbital manifestation across both developed and developing nations [7]. Therefore, it is of interest to report the various eye problems that affect the orbital and anterior segments in people with Type II diabetes mellitus.

## Materials and Methods:

Our current prospective cross-sectional observational study conducted in the department of Ophthalmology over a period of 18 months from June 2022 to December 2023 with 91 patients with thorough history and general examination, past history (diabetes mellitus, ocular trauma, any ocular surgery, COVID 19 infection) and ocular examination (corrected Visual acuity recording, measurement of IOP in both the eye, slit lamp examination, direct and indirect ophthalmoscopy, tests for Dry eye, gonioscopy and Anterior segment optical coherence tomography.

## Inclusion criteria:

[1] Diabetes mellitus of more than 2 year.

[2] Age between 35-70 years.

## Exclusion criteria:

[1] Disorder like connective tissue, thyroid, diabetic retinopathy, diabetic coma

[2] Traumatic corneal opacities

[3] Muscle palsies due to cerebrovascular accident, trauma

[4] Undergone previous ocular surgery

[5] Head injuries

[6] Non-compliant patient

## Statistical analysis:

The average data were subjected to paired t-tests and Pearson correlation analysis across different follow-up intervals and the analysis of qualitative data were conducted employing the Chi-square test and Fisher's Exact Test.

PSC= Posterior Subcapsular Cataract

NS= Nuclear sclerosis

CNP= Cranial Nerve Palsies

## Results:

Table 1 shows that, majority of patients (47%) were in the age group of 46-55 years followed by 35% in the age group of 56-65 years, 10% in the age group of 35-45 years and 8% in the age group of 66-70 years. The mean age of the patients was 54.98± 7.17 years. Table 2 shows that, 65% were male patients and 43% were females, with male preponderance. Table 3 shows that, 43 (43%) patients had Diabetes mellitus for < 5 years while 49 (49%) and 8 (8%) patients had diabetes mellitus for 5-10 years and > 10 years respectively. Table 4 shows that majority of patients were diagnosed with diabetes mellitus after the age of 40 years (92%). Table 5 shows that, 65 patients (65%) also had HT, 4 patients (4%) had Asthma, 4 patients (4%) had COPD and 27 patients (27%) had no other comorbidity.

Table 6 shows that, 20% patients had 6/6-6/12 in right eye and 23% in left eye, 31% patients had 6/18-6/36 in right eye and 24% in left eye while 46% patients had < 6/60 vision in right eye and 53% in left eye. There is no significant difference in visual acuity of right and left eye (p=0.396). Table 7 shows that, most common ocular manifestation is CT (71%) followed by Blepharitis (26%), dry eye (24%), Mucormycosis (12%), Cranial Nerve Palsies (11%), Recurrent changes in refraction (10%), Primary Open Angle Glaucoma (5%), Recurrent stye (4%), Corneal ulcer (3%), Iridocyclitis (3%), Orbital cellulitis (2%), Rubeosis iridis (1%), Neovascular Glaucoma (1%) respectively. Table 8 shows that, 71 cataracts that were observed in patients, cortical cataract was most commonly seen (43.66%), followed by Posterior Sub capsular Cataract which was seen in 27 patients (38.02%), Nuclear sclerosis in 11 patients (15.49%) and snowflake cataract in 2 patients (2.81%). In my study, mean age of patients at which cataract was diagnosed was 55.6 years. Table 9 shows that, mean age of patients with cataract who had Diabetes mellitus for <5 years was 50.7 years while it was 57.7 years and 65 years for patients with cataract who had Diabetes mellitus for 5-10 years and >10 years respectively. Thus, there was a statistically significant positive correlation in mean age of cataract formation between patients with different durations of diabetes (p=0.0001). Table 10 shows that, out of 43 patients with duration of diabetes mellitus < 5 years, 19 patients (20.93%) had Dry Eye. Among 49 patients with duration of diabetes mellitus 5-10 years, 11 patients (22.45%) had Dry Eye and out of 8 patients with duration of Diabetes mellitus >10 years, 5 patients show incidence of dry eye. By using Chi-square test, there was significant association between duration of diabetes and occurrence of dry eye (p=0.026). This suggests that more the duration of Diabetes, higher are the chances of having dry eye syndrome. Table 11 shows that, among the 100 individuals in this study population, 11 patients (11%) were found with Cranial Nerve Palsies. 6th nerve palsy (40%) were more common than third nerve palsy (40%).

## Discussion:

The eyes of Diabetes mellitus patients were studied in a hospital-based prospective study. They found that most of the patients had normal vision in both eyes [8]. Another study showed that the prevalence of dry eye syndrome was 54.3%. Diabetes and dry eyes appear to have a common association. Further studies need to be undertaken to establish an etiologic relationship. However, examination for dry eye should be an integral part of the assessment of diabetic eye disease [9]. Many studies have shown that there is a strong link between Diabetes mellitus and CT [10, 11]. Another study found that, 54% of people with diabetes mellitus had dry eye syndrome. This indicates that for individuals with diabetes, incorporating a screening for dry eye should be considered a routine component of every eye examination [12] Table 12. Includes different comparative studies.

## Conclusion:

It is vital to inform patients about the possible ocular complications connected with diabetes mellitus and urge regular eye exams to prevent future vision loss and possible complications from diabetes mellitus. Refer newly diagnosed patients with diabetes mellitus to an ophthalmologist immediately. Blood glucose levels must be carefully controlled to effectively manage diabetes. Healthy changes to diet and lifestyle are needed. Further, adherence to treatment plans must be constantly checked.

## Figures and Tables

**Table 1:** Age distribution

**Age (in years)**	**N**	**%**
35-45	10	10%
46-55	47	47%
56-65	35	35%
66-70	8	8%
TOTAL	100	100%
MEAN AGE	54.98± 7.17 years	

**Table 2:** Gender distribution

**Gender**	**N**	**%**
Male	58	58
Female	42	42
Total	100	100%

**Table 3:** Diabetes mellitus

**Duration of DM**	**N**	**%**
<5 years	43	43
5-10 years	49	49
>10 years	8	8
Total	100	100

**Table 4:** Age detection of DM

**Age Group**	**N**	**%**
30-40 years	8	8
41-50 years	50	50
51-60 years	39	39
>60 years	3	3
Total	100	100%

**Table 5:** Comorbidity

**Comorbidities**	**N**	**%**
Hypertension(HT)	65	65
Asthma	4	4
COPD	4	4
None	27	27
Total	100	100%

**Table 6:** Visual acuity

**Visual Acuity (VA)**	**Right eye**		**Left eye**		**P Value**
	N	%	N	%	
6/6-6/12	23	23	23	23	P=0.845
6/18-6/36	31	31	24	24	
6/60-1/60	43	43	50	50	
HM to PL	2	2	2	2	
NO PL	1	1	1	1	
Total	100	100%	100	100%	

**Table 7:** Ocular manifestations

**Ocular Manifestations (OM)**	**N**	**%**
Cataract (CT)	71	71
Blepharitis	24	24
Dry eye	24	24
Mucormycosis	12	12
Cranial Nerve Palsies	11	11
Recurrent changes in refraction	10	10
Primary Open Angle Glaucoma	5	5
Recurrent stye	4	4
Corneal ulcer	3	3
Iridocyclitis	3	3
Orbital cellulitis	2	2
Rubeosis iridis	1	1
Neovascular Glaucoma	1	1

**Table 8:** Cataract types

**Type of Cataract**	**N**	**%**
Nuclear Sclerosis(NS)	11	15.49
PSC	27	38.02
Cortical	31	43.66
Snowflake	2	2.81
Total	71	100%

**Table 9:** Duration of diabetes mellitus

**Duration of DM**	**Mean Age of patients (with and without cataract)**	**Mean Age of patients with Cataract**	**p Value**
<5 years	50.16	50.7± 0.77	P=0.0001
5-10 years	57.47	57.7± 1.43	
>10 years	65	65.0± 2	

**Table 10:** Duration of diabetes mellitus

**Duration of DM**	**Incidence of Dry Eye**	**Adequate Tear Film**	**Total**	**% of patients with dry eye**	**P Value**
< 5 years	8	35	43	20.93%	0.026
5-10 years	11	38	49	22.45%	
>10 years	5	3	8	50%	
Total	24	76	100	100%	

**Table 11:** Cranial nerve palsies

**Cranial nerve Palsy (CNP)**	**N**	**M**	**F**
3rd Nerve Palsy	4	3	1
6rd Nerve Palsy	7	4	3
Total	11	7	4

**Table 12:** Different comparative studies

**Investigator**	**Current Study**	**Kathiara *et al.* [13]**	**Sivaraman *et al.* [14]**	**Sarawade *et al.* [15]**	**Deepa [9]**	**Prabhakar *et al.*[16]**
Title of study	Orbital and Anterior segment OM of Type 2 DM	A Study on Manifestations of DM	A study of OM type 2 Diabetes mellitus at tertiary eye care centre in South India	A clinical study on OM of DM	Evaluation of OM in Type 2 DM	A study on OM in patients with DM
Study sample	100	60	500	500	90	820
Type of DM included in the sample	Type 2	Type 1 and 2	Type 2	Type 1 and 2	Type 2	Type 1 and 2
Age group involvement	47 out of 100 patients were between of 46-55 years (47%)	46 out of 60 patients were above 45 years (76.6%).	In majority, 180 patients out of 500 (36 %) from 60 -69 years	In majority, 207 out of 500 (41.4%) were from 51 to 60 yrs	Majority of patients (42.2%) were between 51-60 years	-
Gender involvement	65% male 43% female	56.6% males 43.3% females	52.2% males 47.8% females	55.8% males 44.2% females	54.4% males 45.5% females	-
Comorbidity	Most common disease was HT (65%)	Most common was HT [20 patients (33.3%)]	HT was a predominant systemic disease. They constituted about 298 cases out of 500 patients -59.6%	-	-	-
M/C ocular manifestation	CT most common 71% (71 out 100 patients) Blepharitis- 24% Dry Eye-24% Ocular Mucormycosis- 12% Cranial nerve palsy-11% Recurrent change in refraction- 10% POAG-5% Recurrent stye- 4% Corneal Ulcer-3% Iridocyclitis- 3% Orbital Cellulitis- 2% Rubeosis iridis- 1%	CT most common 58.33% (35 out of 60 patients) Chalazion 1.66% Blepharitis 1.66% Ptosis-3.33% Corneal Ulcer 5% Iridocyclitis 5% Rubeosis iridis-1.66% Hyphaema-1.66% Glaucoma- 5%	CT most common 221 patients out of 500 (42.2%) Hordeolum externum- 1%, Blepharitis-2.4% Chalazion-2% Neovascular glaucoma- 1.6%, POAG-3.2%, POCG-3.8% Keratitis- 4.2%, Ophthalmoplegia 1.4%	CT in 42.2% Recurrent stye-1.2% Recurrent chalazion- 1.6% Blepharitis-1% Transient change of refraction-1.2% POAG-2.6% Neovascular glaucoma-1.2% Orbital cellulitis-0.4% Extraocular muscle palsy-1% Corneal ulcer-0.8%	In this study, 44.4% patients were found to have Cataract Blepharitis-11.11% Recurrent stye-20% Cranial nerve palsies- 16.60% Orbital cellulitis-5.55% Glaucoma-16.6%	CT most common (456 out of 820 cases i.e 58%) Chalazion-2.9% Corneal ulcers-2% Iridocyclitis-1.8% Rubeosis iridis-1% POAG-6.7% PACG-1.1% NVG-1.2% Recurrent change in refraction-12% Cranial nerve palsies- 1%
Other ocular manifestation	NVG- 1%					
